# Reply to Comment on Chen, J., Su, Y., Si, H., Chen, J. Managerial Areas of Construction and Demolition Waste: A Scientometric Review. *Int. J. Environ. Res. Public Health* 2018, *15* (11), 2350, doi:10.3390/ijerph15112350

**DOI:** 10.3390/ijerph16111886

**Published:** 2019-05-28

**Authors:** Jianguo Chen, Yangyue Su, Hongyun Si, Jindao Chen

**Affiliations:** School of Economics and Management, Tongji University, Shanghai 200092, China; jgchen@tongji.edu.cn (J.C.); sihongyun@tongji.edu.cn (H.S.); chenjindao@tongji.edu.cn (J.C.)

Thank you for the comments made by Ho; it is a great honor that our article has attracted attention. We would like to provide feedback to the comment, which focuses on the following five issues:

**Question 1:** The use of search filters. 

**Answer 1:** The search filters advised by Ho seem preferable to ours; however, there is no significant difference between the results of the two search filters (evidence shown in Figure 1). In a comparison of the two retrieval methods, we found that the number of documents retrieved by the two methods was 494 (our search filters) and 493 (Ho’s search filters) by December 31, 2018, and 530 (our search filters) and 529 (Ho’s search filters) documents were retrieved by April 17, 2019. In the original paper, the data (398 articles) was extracted from the Web of Science (WoS) Core collection database (SCI-EXPANDED, SSCI) in May 2018. Ho may not have realized that our retrieval time ranges were different, which led to the incorrect comparison between 496 in the comment and 398 in our original paper. In addition, on the basis of the boundary of management areas of construction and demolition waste (MA-CDW) as defined in the paper, a manual examination of paper titles and abstracts was conducted to exclude the irrelevant papers. Finally, 281 papers were selected for bibliometric analysis, which ensured that all literature studied was about MA-CDW.

**Question 2:** In the original paper, the authors stated that “document type was limited to articles”; however, two reviews were found in “the top ten critical publications in the MA-CDW” in original paper, as shown in Table 1.

**Answer 2:** The scope of the documents co-citation analysis includes not only 281 core articles from WoS but also their references. The top 10 most cited articles presented in Table 2 were from 281 articles and their references; the two reviews are components of 281 articles and their references.

In addition, the cited frequency generated by Citespace may be different from the citations produced by WoS or Google Scholar because the cited frequency produced by WoS or Google Scholar is from the database of WoS or Google Scholar, respectively. Some MA-CDW articles may be cited by publications that are not relevant to construction and demolition waste in WoS or Google Scholar. For example, the article “A two-level network for recycling sand: A case study”, which is referenced in the penultimate paragraph of Ho’s article, the TC2018 in WoS is 247; however, most citations of this article are irrelevant to MA-CDW, such as [1,2,3,4]. As discussed in the paper, 281 papers were selected by manual examination to ensure all papers related to MA-CDW. Therefore, the document co-citation analysis made by Citespace ensures that meaningful citations (i.e., citations which contribute to the development of MA-CDW knowledge) were detected. In contrast, TC2018 employs a different style of analysis for citations. The purpose behind each of these two methods differs.

**Question 3:** The Web of Science Core Collection is designed for researchers to find published literature, not for bibliometric studies. Therefore, using Web of Science Core Collection with an accurate bibliometric method is critical for all researchers.

**Answer 3:** The literature collected in SCI-E and SSCI (components of WoS Core Collection) has undergone rigorous peer review, including more prestigious journals than non-core collections. SCI-E and SSCI articles are more representative than other collections. In practice, more scholars have carried out bibliometric analysis on SCI-E and SSCI documents, such as [5,6,7,8,9]. In addition, several papers of Ho also have used SCI-E databases for literature analysis, such as [10,11,12,13].

**Question 4:** Due to biases from the Web of Science Core Collection, “front page” put forward by Ho’s group as a filter can avoid introducing unrelated articles for analysis.

**Answer 4:** “Front page” is more desirable, but the literature selected in this paper was manually examined to avoid introducing unrelated articles into the analysis. In addition, this method is more accurate and direct than using “front page” to exclude irrelevant articles, which might provide misleading results to readers.

**Question 5:** In addition, using such limited number of papers for a scientometric review inappropriate from a statistical point of view.

**Answer 5:** To date, no research has defined how many articles are suitable for a scientometric review. The number of manual reviews is often less than 100, such as [14,15,16,17]. Moreover, more than 100 articles comprises a heavy workload for manual reviewers. Conducting manual reviews of 281 articles for the purposes of this paper was both time-consuming and complex, thus it is more appropriate to use scientometric analysis. As stated by Yalcinkaya and Singh, the manual review, although insightful, is prone to bias and is limited in its subjective interpretation [18].

I would like to thank Ho again for his comments on this article, which have provided an opportunity to enhance our collective understanding on scientometrics. The method of searching keywords, as detailed in this article, may not be as perfect as Ho’s methods, but there is little difference between the two search results in terms of search number. We carried out a manual examination to avoid irrelevant articles after searching. We chose articles in SCI-E and SCI datasets to introduce high-quality research in the MA-CDW. There remain shortcomings in our article, but they are not as serious as Ho has indicated. In the future, we will be more rigorous when we undertake scientometric research, especially when we choose search filters for analysis.

## Figures and Tables

**Figure 1:**
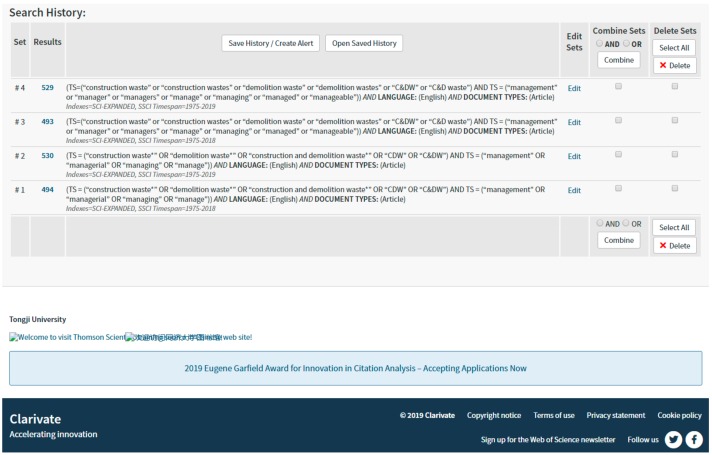
Results of different search filters in Web of Science (WoS).
